# Virological Response to Tenofovir Disoproxil Fumarate in HIV-Positive Patients with Lamivudine-Resistant Hepatitis B Virus Coinfection in an Area Hyperendemic for Hepatitis B Virus Infection

**DOI:** 10.1371/journal.pone.0169228

**Published:** 2016-12-29

**Authors:** Yu-Shan Huang, Sui-Yuan Chang, Wang-Huei Sheng, Hsin-Yun Sun, Kuan-Yeh Lee, Yu-Chung Chuang, Yi-Ching Su, Wen-Chun Liu, Chien-Ching Hung, Shan-Chwen Chang

**Affiliations:** 1 Department of Internal Medicine, National Taiwan University Hospital Hsin-Chu Branch, Hsin-Chu, Taiwan; 2 Department of Laboratory Medicine, National Taiwan University Hospital and National Taiwan University College of Medicine, Taipei, Taiwan; 3 Department of Clinical Laboratory Sciences and Medical Biotechnology, National Taiwan University College of Medicine, Taipei, Taiwan; 4 Department of Internal Medicine, National Taiwan University Hospital and National Taiwan University College of Medicine, Taipei, Taiwan; 5 Department of Parasitology, National Taiwan University College of Medicine, Taipei, Taiwan; 6 Department of Medical Research, China Medical University Hospital, Taichung, Taiwan; 7 China Medical University, Taichung, Taiwan; Kaohsiung Medical University Chung Ho Memorial Hospital, TAIWAN

## Abstract

**Background:**

Sequential addition of tenofovir disoproxil fumarate (TDF) is often needed for patients coinfected with HIV and hepatitis B virus (HBV) who develop HBV resistance to lamivudine after combination antiretroviral therapy (cART) containing only lamivudine for HBV. We aimed to assess the virological response of HBV to add-on TDF in patients coinfected with lamivudine-resistant HBV.

**Methods:**

Between November 2010 and December 2014, 33 HIV/HBV-coinfected patients with lamivudine-resistant HBV and 56 with lamivudine-susceptible HBV were prospectively included. TDF plus lamivudine was used to substitute zidovudine or abacavir plus lamivudine contained in cART in patients with lamivudine-resistant HBV infection, while patients with lamivudine-susceptible HBV infection received TDF plus lamivudine as backbone of cART. Serial determinations of plasma HBV DNA load, HBV serologic markers, and liver and renal functions were performed after initiation of TDF-containing cART.

**Results:**

Of 89 patients included, 38.6% tested positive for HBV envelope antigen (HBeAg) at baseline. The plasma HBV DNA level at enrollment of lamivudine-resistant and lamivudine-susceptible group were 6.1 ± 2.2 log_10_ and 6.0 ± 2.2 log_10_ copies/mL, respectively (p = 0.895). The cumulative percentage of HBV viral suppression in lamivudine-resistant and lamivudine-susceptible group was 81.8% and 91.1% at 48 weeks, respectively (p = 0.317), which increased to 86.7% and 96.2% at 96 weeks, respectively (p = 0.185). At 48 weeks, 11 patients testing HBeAg-positive at baseline failed to achieve viral suppression. In multivariate analysis, the only factor associated with failure to achieve viral suppression at 48 weeks was higher HBV DNA load at baseline (odds ratio, per 1-log_10_ copies/mL increase, 1.861; 95% CI, 1.204–2.878). At 48 weeks, HBeAg seroconversion was observed in 5 patients (1 in the lamivudine-resistant group and 4 in the lamivudine-susceptible group; p = 0.166). During the study period, HBsAg levels decreased over time, regardless of lamivudine resistance. Loss of HBsAg was observed in 3 (3.4%) patients in the lamivudine-susceptible group.

**Conclusions:**

Add-on TDF-containing cART in patients coinfected with lamivudine-resistant HBV achieved a similar rate of HBV viral suppression compared to TDF-containing cART as initial regimen in patients coinfected with lamivudine-susceptible HBV. A higher baseline HBV DNA load and HBeAg positivity were associated with failure to achieve HBV viral suppression.

## Introduction

Hepatitis B virus (HBV) coinfection is common in HIV-positive patients [1]. In Taiwan, 19.8% of HIV-positive patients have concurrent chronic HBV infection [2], though the prevalence of HBsAg positivity has gradually declined after the implementation of nationwide neonatal HBV vaccination program in 1986 [3]. Individuals with both diseases are at greater risks to develop hepatitis and liver decompensation, and their course of chronic HBV infection is more aggressive than those with HBV mono-infection [4–6]. HBV DNA levels have also been shown to predict overall mortality in HIV/HBV-coinfected patients, especially prior to developing acquired immunodeficiency syndrome (AIDS) [7]. To prevent HBV-related liver damage and late complications, it is essential for patients with HBV infection to achieve durable viral suppression before strategies for functional and durable cure of chronic HBV infection are available [8].

Lamivudine that is contained in combination antiretroviral therapy (cART) for HIV used to be the only active antiviral agent against both HIV and HBV. However, the genetic barrier to development of lamivudine resistance is low, as mutations in tyrosine-methionine-aspartate-aspartate (YMDD) motif of HBV emerge frequently. When HIV/HBV-coinfected patients receive lamivudine as the only active drug for HBV, the resistance rates to lamivudine may reach 40% after 2 years and 90% after 4 years in these patients [9–11]. Sequential addition of another anti-HBV agent is often inevitable for HIV/HBV-coinfected patients who started cART in early days before the availability of other anti-HBV agents with greater activity against both HBV and HIV [1].

Among the antiretroviral agents that are active against HIV and HBV, tenofovir disoproxil fumarate (TDF) has potent antiviral effect on both wild-type and lamivudine-resistant HBV [12–14]. TDF-containing cART may lead to high rates of HBV envelope antigen (HBeAg) seroconversion and suppression of HBV replication compared with HBV monotherapy with lamivudine in HIV/HBV-coinfected patients [15]. In HIV/HBV-coinfected patients failing lamivudine, TDF is frequently used as rescue therapy [16, 17]. A previous study reported the association of prior lamivudine exposure with delayed HBV suppression in HIV/HBV-coinfected patients on TDF treatment [18], but this finding was not confirmed in the meta-analysis [19]. To date, data are still limited regarding the impact of prior lamivudine exposure with emergence of lamivudine resistance on the effectiveness of subsequent TDF/lamivudine- or TDF/emtricitabine-containing cART.

In this study, we aimed to assess the virological responses of HBV to TDF-containing cART in HIV/HBV-coinfected patients who had HBV resistance to lamivudine, and to identify factors associated with failure to achieve HBV viral suppression after 48 weeks of treatment with TDF-containing cART.

## Methods

### Patient population and study design

This prospective observational study was conducted at the National Taiwan University Hospital (NTUH), a tertiary care center and the largest designated hospital for HIV care in Taiwan. After TDF and TDF/emtricitabine became available in the clinical care in Taiwan in November 2010 and November 2014, respectively, all adult patients with HBV and HIV coinfection who regularly sought HIV care at NTUH between November 2010 and December 2014 were consecutively included and two groups of HIV/HBV-coinfected patients were defined: add-on TDF to replace one nucleoside reverse-transcriptase inhibitor (NRTI) other than lamivudine in patients who had been taking lamivudine-containing cART with emergence of lamivudine-resistant HBV with (lamivudine-resistant group); and initiation of TDF/lamivudine or TDF/emtricitabine-containing cART in patients who were antiretroviral-naïve or who resumed cART after interruption in the absence of HBV resistance to lamivudine (lamivudine-susceptible group). Patients were excluded if they were aged less than 20 years, their plasma HBV DNA levels were less than 1000 copies/mL within 3 months of enrollment, or they had received or were receiving anti-HBV therapy other than lamivudine such as interferon, telbivudine, entecavir or adefovir. After enrollment, all patients switched to or starting TDF-containing (TDF/lamivudine or TDF/emtricitabine) cART were followed for at least 48 weeks to evaluate the virological response of HBV to TDF-containing cART.

We performed on-treatment analysis to estimate the proportion of patients who achieved undetectable plasma HBV DNA load at each time point. Our primary end-point was the proportion of patients who achieved undetectable plasma HBV DNA load at week 48, and the secondary end-point was the proportion of patients who had undetectable plasma HBV DNA load at week 96. The study was approved by the Research Ethics Committee of NTUH (registration number NTUH-201201028RIB). All patients gave written informed consent before enrollment to provide their blood samples and clinical and laboratory data for research.

### Data collection and definitions

We used a standardized case record form to collect the information on the patients’ demographics, comorbidity, treatment history of cART, abdominal ultrasonography, and laboratory data. The baseline values of plasma HBV DNA load, resistance mutations of HBV, serologies of HBV and HCV, aminotransferase levels, serum creatinine, serum alpha-fetoprotein, CD4 lymphocyte count, and plasma HIV RNA load were obtained within 3 months before initiation of TDF-containing cART. After starting TDF-containing cART, serial blood samples were collected from patients at weeks 4, 8, 12, 24, 36, and 48 and then every 48 weeks subsequently. Serial determinations were performed for plasma HBV DNA load, HBV serologic markers, alpha-fetoprotein, and liver and renal functions. The numbers of patients with HBeAg seroconversion, loss of HBV surface antigen (HBsAg), and HBsAg seroconversion were documented. The estimated glomerular filtration rate (eGFR) was calculated by the modification of diet in renal disease (MDRD) equation.

Undetectable plasma HBV DNA load was defined as <128 copies/mL. Chronic HBV coinfection was defined as the persistence of HBsAg for >6 months, and hepatitis C coinfection was defined by positive anti-HCV antibody. Hepatitis flare was defined as >5-fold elevation of serum aspartate aminotransferase (AST) and alanine aminotransferase (ALT) levels (upper limit of normal for AST and ALT, 36 and 41 IU/L, respectively); and hyperbilirubinemia as a total bilirubin >2.0 mg/dL without evidence of hemolysis or taking atazanavir-containing cART. We used AST-to-platelet ratio index (APRI) for the noninvasive evaluation of liver fibrosis. The APRI was determined as follows: [(AST/upper limit of normal of AST)/platelet count (10^9^/L)] x 100. Parenchymal liver disease or cirrhosis was documented by the presence of coarse echogenicity and irregular liver surface as demonstrated by abdominal ultrasonography.

### Laboratory investigations

#### Determinations of plasma HBV DNA load and HBV serologic markers

Plasma HBV DNA load was quantified using the Abbott Real Time HBV assay (Abbott Laboratories, Abbott Park, IL, U.S.A) with a lower detection limits of 15 IU/mL after 2.5-fold dilution of serum samples, and the results were stated as 1 IU/mL = 3.41 copies/mL. HBeAg, anti-HBe antibody, HBsAg, anti-HBs antibody, and antibodies to HCV were determined using enzyme immunoassay (Abbott Laboratories, Abbott Park, IL, U.S.A). Quantification of HBsAg levels was determined by ARCHITECT il000 chemiluminescence microparticle immunoassay (Abbott Laboratories, Abbott Park, IL, U.S.A).

#### HBV genotyping and detection of lamivudine-resistant mutations

A nested PCR was performed to amplify part of the polymerase gene containing the tyrosine-methionine-aspartate-aspartate (YMDD) motif from patients with detectable HBV DNA. The expected size of PCR product is 1.2kb. The first and second primer pairs used are 1821F (5'-TTT TTC CCC TCT GCC TAA TCA-3')/1825R (5'-AAA AAG TTG CAT GGT GCT GG-3') and 3106F (5'- ACA CTG CCA GCA GCA CCT CCT CC -3')/1088R (5'-AGC CTG CTT AGA TTG TAT ACA TGC-3'). The amplification condition was 35 cycles of 94°C for 30s, 56°C for 30s, 72°C for 2 min, and a final extension at 72°C for 7 min. A 5-μL aliquot of the first round PCR product was used for the second round PCR, whose condition was the same as the first round. Sequencing analysis was performed with an automatic ABI-DNA sequencer (Model 377 A; Applied Biosystems). The HBV genotype was determined by constructing the phylogenetic trees using the neighbor-joining method and the Kimura 2-parameter distance matrix listed in the MEGA (molecular evolutionary genetics analysis) analytical package [20]. The presence of the YMDD mutant (rt pol gene mutations rtM204V plus rtM204I) and/or rtL180M was confirmed by sequencing readout.

#### Determinations of plasma HIV RNA load CD4 lymphocyte count

Plasma HIV RNA load was quantified using the Cobas AmpliPrep/Cobas TaqMan HIV-1 test (version 2.0, Roche Molecular Systems, Inc.). CD4 lymphocyte count was determined using flow cytometry (BD FACS Calibur, Becton Dickinson and Coulter Epics XL, Beckman Coulter, CA, USA).

### Statistical analysis

Patients’ demographics and basic characteristics were evaluated by descriptive statistics. Categorical variables were compared using chi-square test or Fisher’s exact test. Continuous variables were compared using the Kruskal-Wallis one-way analysis of variance or Mann-Whitney U test. For data from correlated-samples, variables were compared using Wilcoxon signed rank test. A two-tailed p value <0.05 was considered statistically significant. Univariate and multivariate logistic regression model was used to assess factors associated with failure to achieve viral suppression after 48 weeks of TDF-containing cART treatment. Stepwise model selection with Akaike Information Criterion (AIC) was performed, and variables were entered into the model with p value <0.25 as a requirement for acceptance. All statistical analyses were performed using SPSS software version 20.0 (SPSS Inc., Chicago, IL, USA).

## Results

### Clinical characteristics of patients

During the 4-year study period, a total of 89 HIV/HBV-coinfected patients were included for analysis. Among them, 33 patients had coinfection with lamivudine-resistant HBV, and 56 patients had lamivudine-susceptible HBV. The baseline characteristics of the patients are shown in Table 1. The majority of patients were middle-aged men who have sex with men. Genotype B was the dominate HBV subtype in both groups, followed by genotype C. Patients with lamivudine-resistant HBV had been exposed to cART containing only lamivudine for HBV for an average of 6.5 years before enrollment, and all patients with lamivudine-susceptible HBV were cART-naïve, except one who had interrupted cART (15 months of abacavir/lamivudine plus atazanavir) for 4 years before enrollment.

**Table 1:** Clinical characteristics of 89 HIV-positive patients coinfected with lamivudine-resistant or lamivudine-susceptible hepatitis B virus

	Patients with LAM-R HBV (n = 33)	Patients with LAM-S HBV (n = 56)	*P* value
Age, years	42 ± 8	36 ± 8	0.001
Male sex	33 (100)	55 (98.2)	0.999
Years since HIV diagnosis	12.8 ± 4.7	5.2 ± 2.9	<0.001
HBV genotype			
B	28/33 (84.8)	37/43 (86.0)	0.999
C	5/33 (15.2)	6/43 (14.0)	
No data	0	13	
Previous LAM use, years	6.5 ± 3.9	NA	
Positive HBeAg at baseline	16/33 (48.5)	18/55 (32.7)	0.142
HBsAg level at baseline, log_10_ IU/mL	5.3 ± 2.1 (n = 33)	3.5 ± 1.0 (n = 38)	<0.001
Plasma HBV DNA level at enrollment, log_10_ copies/mL	6.1 ± 2.2	6.0 ± 2.2	0.895
3–5 log_10_ copies/mL	14 (42.4)	23 (41.1)	
>5 log_10_ copies/mL	19 (57.6)	33 (58.9)	
Hepatitis flares within the preceding one year of enrollment	7 (21.2)	NA	
ALT at baseline, IU/L	56 ± 55	52 ± 48	0.383
APRI score at baseline	0.5 ± 0.4	0.9 ± 1.4	0.099
Cirrhosis or parenchymal liver disease at baseline	10/31 (32.2)	10/39 (25.6)	0.543
Chronic HCV infection at baseline	0 (0)	2 (3.6)	
CD4 cell count at baseline, cells/μl	552 ± 382	249 ± 220	<0.001
Plasma HIV RNA load at baseline, log_10_ copies/mL,	1.7 ± 0.6	4.9 ± 0.6	<0.001
Plasma HIV RNA load <200 copies/mL at baseline	31 (93.9)	0 (0)	
NRTI backbone before tenofovir and lamivudine			
Zidovudine/lamivudine	10 (30.3)	NA	
Abacavir/lamivudine	22 (66.7)	NA	
Didanosine and lamivudine	1 (3)	NA	
NNRTI-based cART	15 (45.5)	39 (69.6)	0.024
PI-based cART	18 (54.5)	14 (25)	0.005
II-based cART	0 (0)	3 (5.4)	
Follow-up duration, weeks,	202 ± 58	147 ± 54	<0.001

Results are *n* (%), or mean ± standard deviation.

**Abbreviations:** ALT, alanine aminotransferase; APRI, AST-to-platelet ratio index; cART, combination antiretroviral therapy; HBV, hepatitis B virus; HBeAg, HBV envelope antigen; HBsAg, HBV surface antigen; HCV, hepatitis C virus; II, integrase inhibitor; LAM, lamivudine; LAM-R, LAM-resistant; LAM-S, LAM-susceptible; NA, not applicable; NNRTI, non-nucleoside reverse transcriptase inhibitors; NRTI, nucleos(t)ide reverse transcriptase inhibitors; PI, protease inhibitor.

While the two groups of patients had similar mean HBV DNA loads (6.1 ± 2.2 vs. 6.0 ± 2.2 log_10_ copies/mL, p = 0.895) and HBeAg-positive rates (48.5% vs. 32.7% in patients with available data, p = 0.142) at baseline, the two groups differed significantly in many clinical characteristics because the great majority of patients in the lamivudine-susceptible group were antiretroviral-naïve. Compared with patients in the lamivudine-susceptible group, patients in the lamivudine-resistant group had a higher mean age (p = 0.001), HBsAg titer (p<0.001), and CD4 cell count (p<0.001), a lower mean plasma HIV RNA load (p<0.001) and higher proportion of patients with baseline HIV RNA load <200 copies/mL (lamivudine-resistant vs. lamivudine-susceptible, 93.9% vs. 0%). Patients with HBeAg positivity had higher baseline and serial HBV DNA compared with those with HBeAg negativity (S1 Table).

### Virological response of HBV to tenofovir-containing antiretroviral therapy

**Fig 1:**
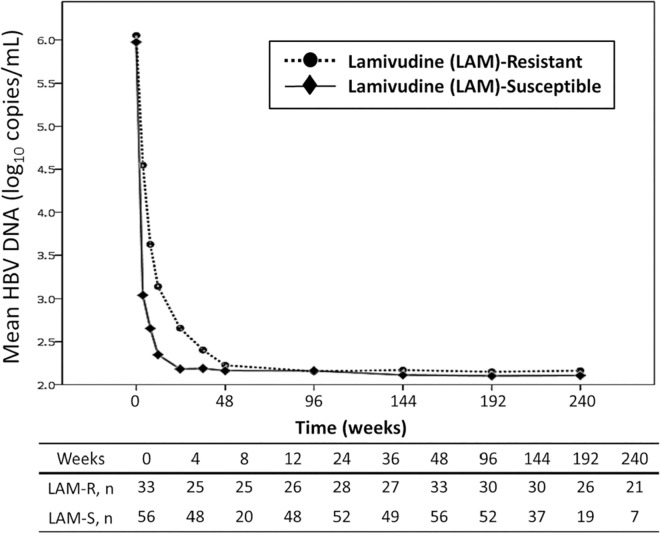
Changes of plasma HBV DNA load in HIV/HBV-coinfected patients with lamivudine-resistant HBV (n = 33) or lamivudine-susceptible HBV (n = 56) who were on tenofovir-containing combination antiretroviral therapy

**Fig 2:**
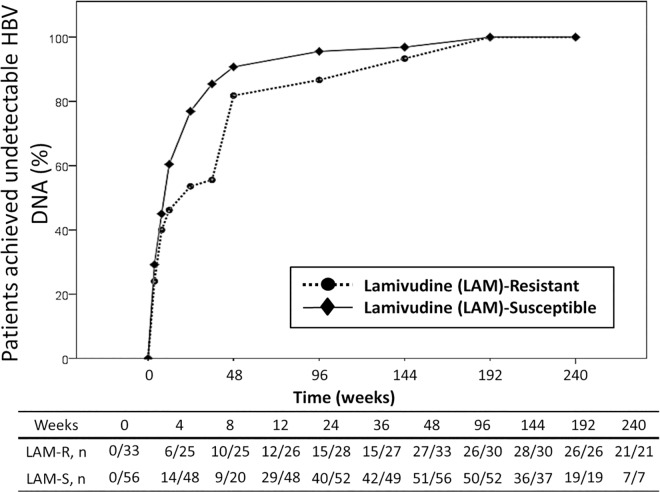
Cumulative percentage of HIV/HBV-coinfected patients with lamivudine-resistant HBV (n = 33) or lamivudine-susceptible HBV (n = 56) who had achieved undetectable HBV DNA (<128 copies/mL) during the follow-up period of tenofovir-containing combination antiretroviral therapy

The average follow-up duration of the two groups of patients was 167 weeks. Fig 1 shows the mean changes of plasma HBV DNA load from baseline over time. The mean changes of plasma HBV DNA load from baseline were -2.2, -2.9, -2.8, -3.7, -4.0, -3.8, and -4.0 log_10_ copies/mL in patients with lamivudine-resistant HBV, and -2.5, -3.4, -3.2, -3.6, -3.9, -3.8, and -3.9 log_10_ copies/mL in patients with lamivudine-susceptible HBV at weeks 4, 8, 12, 24, 36, 48 and 96, respectively. The mean plasma HBV DNA loads of the two groups were similar at 48 weeks and 96 weeks (p = 0.169 and p = 0.351, respectively) (S2 Table). The cumulative percentage of patients who achieved undetectable plasma HBV viral load (<128 copies/mL) in patients with lamivudine-resistant HBV and those with lamivudine-susceptible HBV was 81.8% (27 of 33 patients) and 91.1% (51 of 56 patients) at 48 weeks, respectively (p = 0.317), which increased to 86.7% (26 of 30 patients) and 96.2% (50 of 52 patients) at 96 weeks, respectively (p = 0.185) (Fig 2). At 48 weeks, 11 patients did not achieve undetectable HBV DNA load. No resistance mutations to TDF were found in these patients, however. The mean CD4 count of all patients was 499 ± 503 cells/μl at week 48 and 521 ± 269 cells/μl at week 96. The mean plasma HIV RNA load of all patients was 1.6 ± 0.3 log_10_ copies/mL at week 48 and 1.5 ± 0.1 log_10_ copies/mL at week 96.

**Fig 3:**
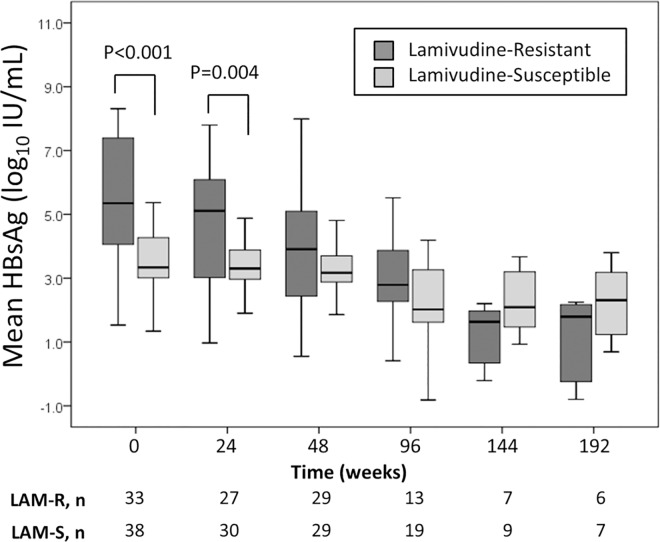
Changes of HBsAg levels in HIV/HBV-coinfected patients with lamivudine-resistant HBV or lamivudine-susceptible HBV on tenofovir-containing combination antiretroviral therapy

The changes of HBsAg levels with time are presented in Fig 3. Declines of mean HBsAg levels were observed in both groups of patients during the first 48 weeks of TDF-containing cART. Compared with patients in the lamivudine-resistant group, patients in the lamivudine-susceptible group had lower HBsAg levels at baseline (p<0.001) and at 48 weeks (p = 0.070), though the difference at week 48 did not reach statistical significance.

The changes of plasma HBV DNA load and HBsAg levels with treatment with TDF plus emtricitabine or lamivudine in patients with HBeAg positivity and those with HBeAg negativity are shown in S3 Table and S1–S3 Figs.

Loss of HBsAg was observed in 3 (3.4%) patients at 96 weeks (n = 2) and 144 weeks (n = 1), and one of them had HBsAg seroconversion. All of these 3 patients were in the lamivudine-susceptible group. Another one patient in the lamivudine-susceptible group was found to have both positive HBsAg and anti-HBs at 144 weeks. At 48 weeks, HBeAg seroconversion was observed in 1 of 16 (6.3%) patients in the lamivudine-resistant group, and 4 of 16 (25%) patents in the lamivudine-susceptible group (p = 0.166).

### Factors associated with failure to achieve viral suppression at 48 weeks

Overall, 78 (87.6%) patients had achieved undetectable HBV DNA load before 48 weeks. All 11 patients who failed to achieve undetectable HBV DNA load at 48 weeks had positive HBeAg at baseline, compared with only 23 (29.9%) in 77 patients that had successful HBV viral suppression (1 patients did not have HBeAg data at baseline). In univariate analysis, the factors associated with failure to achieve viral suppression at 48 weeks were higher HBV DNA and HBsAg levels at baseline (p = 0.004 and p = 0.009, respectively; Table 2). In multivariate analysis, the only independent factor associated with failure to achieve viral suppression at 48 weeks was higher plasma HBV DNA load at baseline (adjusted odds ratio, per 1-log_10_ copies/mL increase, 1.861; 95% CI, 1.204–2.878; p = 0.005).

**Table 2:** Multivariate logistic analysis to identify the factors associated with failure to achieve HBV viral suppression (<128 copies/ mL) after 48 weeks of tenofovir-containing antiretroviral therapy

Variable^a^	Achieve viral suppression (n = 78)	Failure to achieve viral suppression (n = 11)	Logistic Regression			
			OR (95% CI)	Univariate p =	OR (95% CI)	Multivariate p =
Age, years	39 ± 8	34 ± 7	0.931 (0.853–1.016)	0.107	0.900 (0.806–1.003)	0.058
HBV genotype B	56/65 (86.2)	9/11 (81.8)	0.723 (0.134–3.904)	0.706		
Lamivudine resistance of HBV	27 (34.6)	6 (54.5)	2.267 (0.633–8.113)	0.208	4.429 (0.894–21.946)	0.068
CD4 count at baseline, cells/μl	360 ± 334	375 ± 251	1.000 (0.998–1.002)	0.884		
Plasma HIV RNA load at baseline, log_10_ copies/mL	3.8 ± 1.6	3.1 ± 1.6	0.770 (0.521–1.139)	0.191		
Plasma HBV DNA load at baseline, log_10_ copies/mL	5.7 ± 2.1	8.1 ± 1.3	1.875 (1.223–2.874)	0.004	1.861 (1.204–2.878)	0.005
HBsAg level at baseline, log_10_ IU/mL	4.1 ± 1.8 (n = 61)	5.8 ± 1.5 (n = 10)	1.680 (1.141–2.474)	0.009		
Positive HBeAg at baseline^b^	23/77 (29.9)	11/11 (100)				

Results are *n* (%), or mean ± standard deviation.

**Abbreviations:** HBV, hepatitis B virus; HBeAg, HBV envelope antigen; HBsAg, HBV surface antigen; CI, confidence interval; OR, odds ratio.

^a^Variables considered for entry into multivariate logistic regression model included variables with P values <0.25 in univariate analysis.

^b^ All patients failing to achieve viral suppression had positive HBeAg at baseline and, therefore, the factor was not included in the logistic regression.

### Adverse events on TDF-containing treatment and patient outcomes

The mean baseline eGFR of patients with lamivudine-resistant HBV and those with lamivudine-susceptible HBV were 97 ml/min/1.73m^2^ and 110 ml/min/1.73m^2^, respectively, and 98 ml/min/1.73m^2^ and 102 ml/min/1.73m^2^ at week 48, respectively. The serial data of eGFR are shown in S4 Table. During the study period, one patient in the lamivudine-resistant group discontinued TDF because her eGFR decreased from 37 ml/min/1.73m^2^ at baseline to 12 ml/min/1.73m^2^ at week 36. After TDF was discontinued, renal function improved and the patient did not have hepatitis flares until study ended. No other TDF-related severe adverse events were documented. Two patients experienced acute hepatitis resulting from acute HCV infection and trimethoprim/sulfamethoxazole-associated hepatotoxicity, respectively. Seven patients were lost to follow-up after one year, one patient died from lung cancer, and the remaining patients continued their HIV care regularly. With TDF-containing cART, the ALT levels and APRI scores were significantly lower at week 48 (ALT, p<0.001; APRI, p = 0.007) and week 96 (ALT, p = 0.001; APRI, p<0.001) compared with the baseline data (S5 Table).

## Discussion

This study demonstrates that virological response of HBV to subsequent add-on TDF to replace one NRTI of current cART in HIV/HBV-coinfected patients with emergence of lamivudine-resistant HBV was similar to that of initiation of TDF-containing cART as the first regimen in patients without lamivudine-resistant HBV. Among HIV/HBV-coinfected patients, the factors associated with failure to achieve HBV viral suppression at 48 weeks were a higher plasma HBV DNA load and HBeAg positivity prior to TDF-containing cART. During study period, HBsAg levels decreased over time, regardless of the presence or absence of lamivudine resistance.

To prevent complications of chronic HBV infection, the current goal of HBV therapy is to achieve sustained suppression of viral replication to the lowest detectable level. In antiretroviral-naïve HIV/HBV-coinfected patients, TDF/lamivudine- or TDF/emtricitabine-based regimens are recommended as first-line cART [21]. Dual therapy with TDF/lamivudine or TDF/emtricitabine against HBV not only prevents the emergence of lamivudine resistance [22], but also suppresses HBV replication more effectively than lamivudine monotherapy [15, 23, 24]. However, studies are limited that explore the activity of add-on TDF on HBV replication in HIV/HBV-coinfected patients who fail previous lamivudine monotherapy for HBV [16, 17, 25, 26], including those with advanced liver disease [27]. In 2006, Schmutz et al. published a 1:2 matched pair analysis comparing the antiviral efficacy of first-line treatment with TDF and lamivudine to sequential TDF after the development of HBV-DNA relapse (>10^5^ copies/mL) while on lamivudine monotherapy [16]. They concluded that the proportions of patients with undetectable plasma HBV DNA load at 12, 48 or 96 weeks and loss of HBeAg and HBsAg were similar between the two study arms. However, not every patient in the study was assessed for lamivudine resistance before switching to add-on TDF. A later study investigating the long-term efficacy of TDF showed no difference of virological response (HBV DNA <20 IU/mL) up to 60 months between HIV/HBV-coinfected patients with or without lamivudine resistance at baseline [28].

In line with those previous reports, we found that similar proportions of patients who achieved undetectable HBV DNA load at 48 weeks and 96 weeks of treatment between the patients with and those without lamivudine-resistant HBV. While more studies of long-term clinical and virological outcomes of the two groups of patients are warranted, the findings support the use of TDF/lamivudine- or TDF/emtricitabine-based therapy in HIV/HBV-coinfected patients who develop lamivudine resistance of HBV. For HIV/HBV-coinfected patients currently on lamivudine monotherapy for HBV with good suppression of HBV replication, whether it is more beneficial to initiate TDF-based cART immediately or to continue lamivudine monotherapy for HBV with careful HBV load monitoring remains uncertain.

In our study, a higher HBV DNA load and HBeAg positivity at baseline were associated with failure to achieve HBV suppression at 48 weeks. These two factors have also been shown to influence time to accomplish virological response in TDF-experienced, HBV mono-infected patients with lamivudine failure [29]. As in the meta-analysis [19], we did not find the association between lamivudine exposure and delayed HBV suppression. Another study from Kosi et al [15] that compared the effect of different anti-HBV regimens contained in cART (lamivudine monotherapy, simultaneous TDF/lamivudine, and lamivudine followed by TDF/lamivudine) revealed no correlation between anti-HBV treatment options and HBV viral suppression. Kosi et al described that HBV genotype non-A, detectable HIV viremia at 1 year, lower CD4 count, and reporting <95% adherence were significant risk factors for HBV virological non-response [15]. Our patients in this study were only infected with HBV genotypes B and C, which were the dominant genotypes among Taiwanese patients [30], and no difference in HBV suppression was found between patients with either genotype. In contrast, plasma HIV RNA load and CD4 count prior to TDF therapy had no impact on HBV viral suppression in our study.

Along with HBV virological suppression, HBeAg seroconversion and HBsAg loss are also important therapeutic goals in HBV treatment. In therapeutic trials of lamivudine- or TDF-containing cART, HBeAg seroconversion rates among HIV/HBV-coinfected patients ranged from 17 to 46% after 2 to 5 years of cART [28, 31–33]. In our study, 5 of 32 (15.6%) patients with available data had HBeAg seroconversion at week 48. Although no statistical significance was demonstrated due the small sample size, a higher HBeAg seroconversion rate was observed in the lamivudine-susceptible group than the lamivudine-resistant group (25% vs. 6.3%, p = 0.166).

HBsAg loss was generally uncommon in HIV/HBV-coinfected patients. An annual HBsAg seroconversion rate of 2.6% in HIV/HBV-coinfected patients has been reported [34]. In our cohort, we found that 3.4% of patients experienced HBsAg loss. The study by Matthews et al demonstrated a higher rate (13%) of HBsAg loss over a median follow-up of 108 weeks [35]. The author suggested that the higher rate of HBsAg loss might result from immune restoration, represented by marked CD4 count elevation and sustained HIV suppression in their study. In HIV/HBV-coinfected populations, studies exploring quantitative HBsAg kinetics were still limited. Declines in quantitative HBsAg had been shown to predict HBsAg seroclearance [32, 36]. Our study demonstrated decreasing trends of quantitative HBsAg up to 96 weeks in response to TDF-containing cART. However, the number of patients with HBsAg loss is too small in our cohort for further investigation into the correlation between HBsAg titer and HBsAg seroclearance.

Our study has several limitations. First, the sample size is small and, therefore, we were not able to demonstrate a statistically significant difference of virological response rates of lamivudine-resistant and lamivudine-susceptible HBV to TDF-containing cART between the two groups. With introduction of TDF for HBV or HIV infection, HIV/HBV-coinfected patients start cART containing TDF/lamivudine or TDF/emtricitabine according to the HIV treatment guidelines [21]; it is therefore that resistance of HBV to lamivudine have become rare for such comparisons to conduct. Second, the study was not a randomized clinical trial and the two groups of patients with significant differences in several baseline characteristics were included in routine clinical care in different time periods when cART has evolved significantly. Third, we did not evaluate the adherence of patients. The failure of HBV viral suppression may result from non-adherence to cART. However, all 11 patients who failed to achieve HBV suppression at 48 weeks in our study had plasma HIV RNA loads ranging from undetectable to 90 copies/mL, suggesting good adherence to cART.

In conclusion, add-on TDF-containing cART in HIV/HBV-coinfected patients with lamivudine-resistant HBV achieved a similar rate of HBV viral suppression compared to initiation of TDF-containing cART in HIV/HBV-coinfected patients with lamivudine-susceptible HBV as the frontline regimen for both HIV and HBV. A higher plasma HBV DNA load and HBeAg positivity at baseline were associated with failure to achieve HBV viral suppression at week 48 of TDF-containing cART. For HIV/HBV-coinfected patients failing lamivudine, TDF combined with lamivudine or emtricitabine could serve as an effective and well-tolerated therapy against HBV.

## Supporting Information

S1 Fig Changes of plasma HBV DNA load in HIV/HBV-coinfected patients with baseline positive HBeAg (n = 34) or negative HBeAg (n = 54) who were on tenofovir-containing antiretroviral therapy. (TIF)

S2 Fig Cumulative percentages of HIV/HBV-coinfected patients with baseline positive HBeAg (n = 34) or negative HBeAg (n = 54) who had achieved undetectable HBV DNA (<128 copies/mL) during the follow-up period of tenofovir-containing antiretroviral therapy. (TIF)

S3 Fig Changes of HBsAg level in HIV/HBV-coinfected patients with baseline positive HBeAg (n = 34) or negative HBeAg (n = 54) who were on tenofovir-containing antiretroviral therapy. (TIF)

S1 Table Clinical characteristics of 88 HIV/HBV-coinfected patients with HBeAg-positive or -negative at baseline. (DOC)

S2 Table Response of HBV to tenofovir-containing combination antiretroviral therapy based on presence of lamivudine-resistance to HBV. (DOC)

S3 Table Response of HBV to tenofovir-containing antiretroviral therapy based on presence of baseline HBeAg positivity. (DOC)

S4 Table Change of estimated glomerular filtration rate (eGFR) under tenofovir-containing combination antiretroviral therapy based on presence of lamivudine-resistance to HBV. (DOC)

S5 Table Changes of alanine aminotransferase, alpha-fetoprotein, and APRI score in response to tenofovir-containing combination antiretroviral therapy based on presence of lamivudine-resistance to HBV. (DOC)
