# Supplementary figures and images for: Virological Response to Tenofovir Disoproxil Fumarate in HIV-Positive Patients with Lamivudine-Resistant Hepatitis B Virus Coinfection in an Area Hyperendemic for Hepatitis B Virus Infection
Source: PLoS One. 2016 Dec 29;11(12):e0169228. doi: 10.1371/journal.pone.0169228 (PMC5199102; doi:10.1371/journal.pone.0169228)

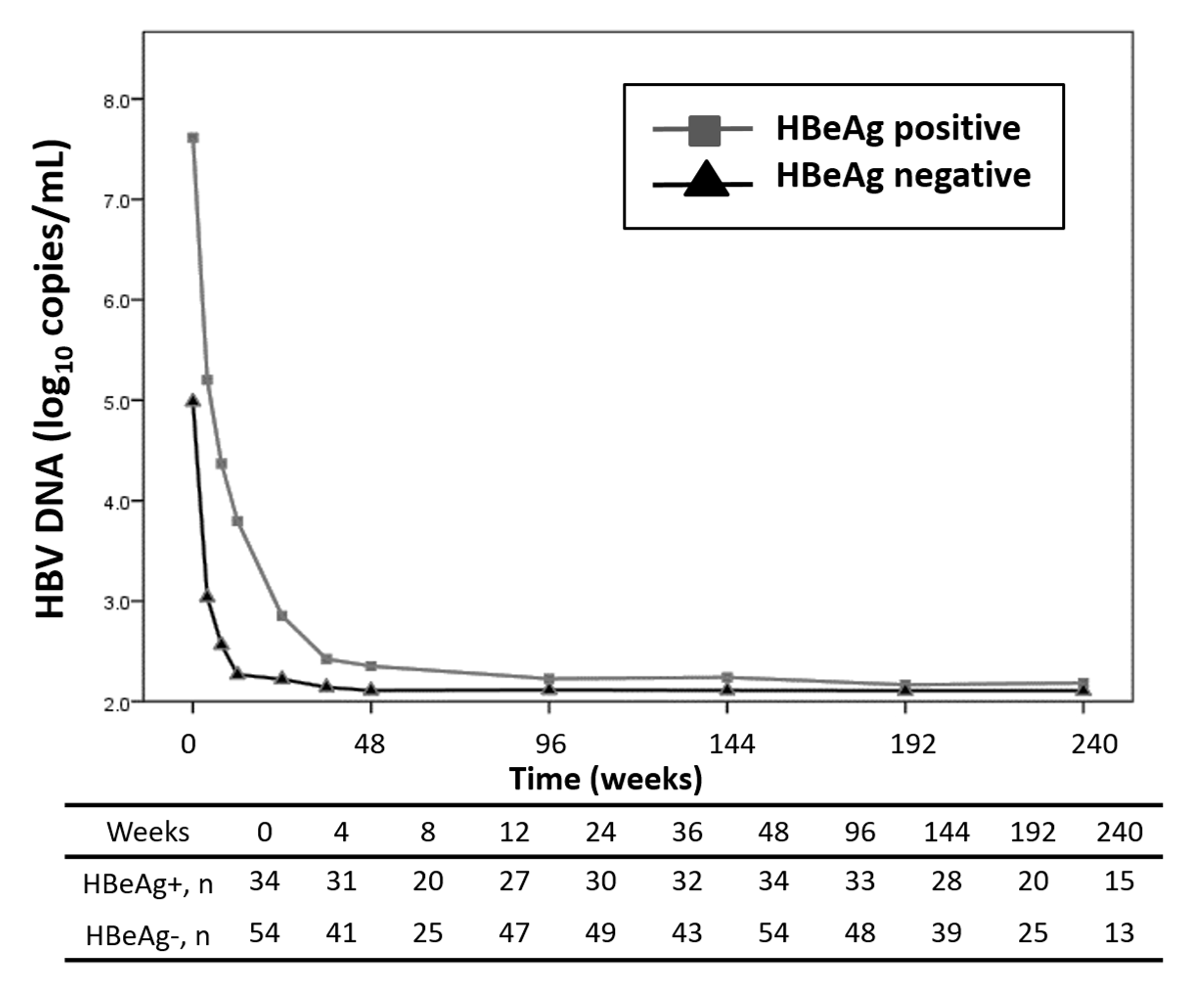

Supplement: S1 Fig — (TIF) [file pone.0169228.s001.tif]

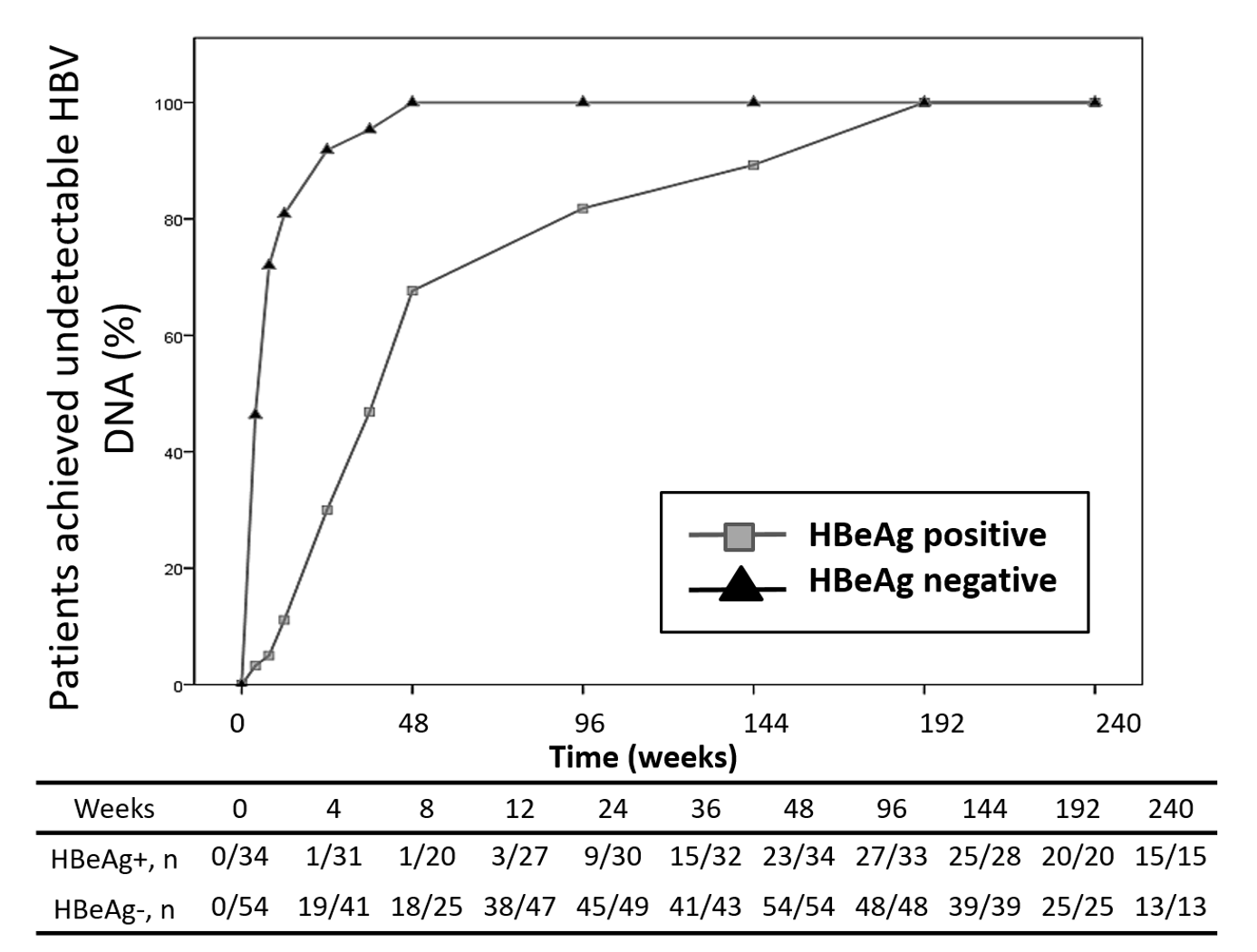

Supplement: S2 Fig — (TIF) [file pone.0169228.s002.tif]

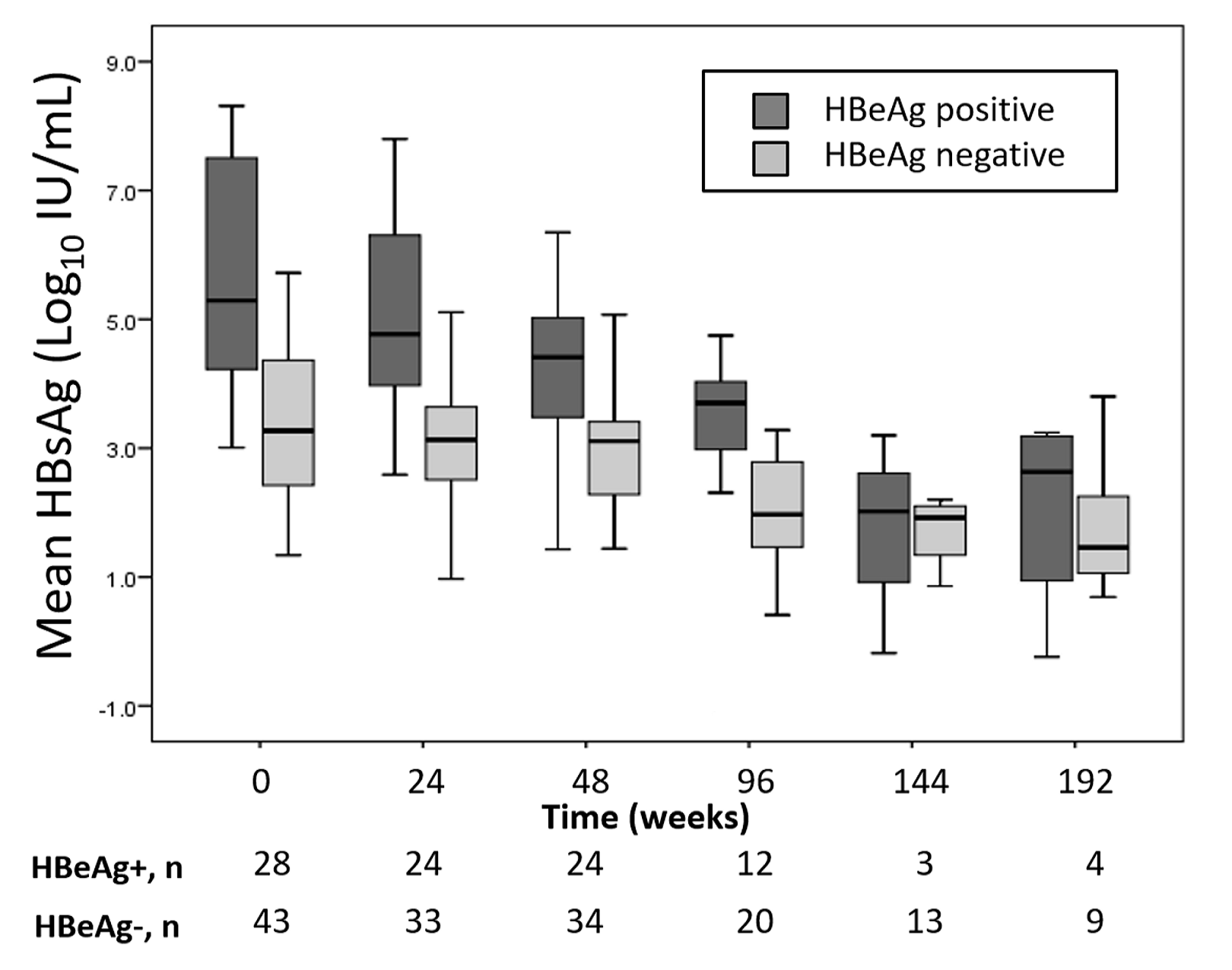

Supplement: S3 Fig — (TIF) [file pone.0169228.s003.tif]
