# Supplementary material for: Virological Response to Tenofovir Disoproxil Fumarate in HIV-Positive Patients with Lamivudine-Resistant Hepatitis B Virus Coinfection in an Area Hyperendemic for Hepatitis B Virus Infection
Source: PLoS One. 2016 Dec 29;11(12):e0169228. doi: 10.1371/journal.pone.0169228 (PMC5199102; doi:10.1371/journal.pone.0169228)
Supplement: S1 Table — (DOC) [file pone.0169228.s004.doc]

**Supplementary Table 1** Clinical characteristics of 88 HIV/HBV-coinfected patients with HBeAg-positive or -negative at baseline

|  | Patients with positive HBeAg at baseline  (n=34) | Patients with negative HBeAg at baseline  (n=54) | p value |
| --- | --- | --- | --- |
| Age, years | 35.7 ± 7.8 | 39.5 ± 8.3 | 0.022 |
| Male sex | 33 (97.1) | 54 (100) | 0.386 |
| Years since HIV diagnosis | 8.8 ± 4.8 | 7.6 ± 5.5 | 0.092 |
| HBV genotype |  |  |  |
| B | 25/34 (73.5) | 40/54 (74.1) | 0.340 |
| C | 6/34 (17.6) | 5/54 (9.3) |  |
| No data | 3 | 9 |  |
| Previous lamivudine use, years | 5.7 ± 3.7 | 7.3 ± 4.0 | 0.195 |
| Presence of lamivudine-resistant HBV | 16/34 (47.1) | 17/54 (31.5) | 0.142 |
| HBsAg level at baseline, log_10_ IU/mL | 5.6 ± 1.6 (n=28) | 3.5 ± 1.4 (n=43) | <0.001 |
| Plasma HBV DNA level at enrollment, log_10_ copies/mL | 7.6 ± 1.6 | 5.0 ± 1.8 | <0.001 |
| 3-5 log_10_ copies/mL | 4 (11.8) | 21 (38.9) |  |
| >5 log_10_ copies/mL | 30 (88.2) | 33 (61.1) |  |
| ALT at baseline, IU/L | 55 ± 55 | 52 ± 49 | 0.349 |
| APRI score at baseline | 0.6 ± 0.4 | 0.9 ± 1.4 | 0.773 |
| Cirrhosis or parenchymal liver disease at baseline | 5/27 (18.5) | 15/42 (35.7) | 0.124 |
| Chronic HCV infection at baseline | 1(2.9) | 1(1.9) | 0.738 |
| CD4 cell counts at baseline, cells/μl, | 399 ± 260 | 343 ± 360 | 0.087 |
| Plasma HIV RNA load at baseline, log_10_ copies/mL, | 3.3 ± 1.6 | 3.9 ± 1.6 | 0.109 |
| Plasma HIV RNA load <200 copies/mL at baseline | 15 (44.1) | 16 (29.6) |  |
| NNRTI-based cART | 21 (61.8) | 33 (61.1) | 0.951 |
| PI-based cART | 13 (38.2) | 18 (33.3) | 0.639 |
| II-based cART | 0 (0) | 3 (5.6) |  |
| Follow-up duration, weeks | 184 ± 60 | 159 ± 63 | 0.076 |

Results are *n* (%), or mean ± standard deviation.

**Abbreviations:** ALT, alanine aminotransferase; APRI, AST-to-platelet ratio index; cART, combination antiretroviral therapy; HBV, hepatitis B virus; HBeAg, HBV envelope antigen; HBsAg, HBV surface antigen; HCV, hepatitis C virus; II, integrase inhibitor; NA, not applicable; NNRTI, non-nucleoside reverse transcriptase inhibitors; NRTI, nucleos(t)ide reverse transcriptase inhibitors; PI, protease inhibitor
