# Supplementary material for: Virological Response to Tenofovir Disoproxil Fumarate in HIV-Positive Patients with Lamivudine-Resistant Hepatitis B Virus Coinfection in an Area Hyperendemic for Hepatitis B Virus Infection
Source: PLoS One. 2016 Dec 29;11(12):e0169228. doi: 10.1371/journal.pone.0169228 (PMC5199102; doi:10.1371/journal.pone.0169228)
Supplement: S2 Table — (DOC) [file pone.0169228.s005.doc]

**Supplementary Table 2**. Response of HBV to tenofovir-containing combination antiretroviral therapy based on presence of lamivudine-resistance to HBV

|  | Plasma HBV DNA (log_10_ copies/mL) | | | | | |  | Plasma HBV DNA <128 copies/mL, n (%) | | | |  | | HBsAg level (log_10_ IU/mL) | | | | |
| --- | --- | --- | --- | --- | --- | --- | --- | --- | --- | --- | --- | --- | --- | --- | --- | --- | --- | --- |
|  | LAM-R | n | LAM-S | n | | p |  | LAM-R | LAM-S | p |  | | LAM-R | | n | LAM-S | n | p |
| Baseline | 6.1 ± 2.2 | 33 | 6.0 ± 2.2 | 56 | 0.895 | |  | 0/33 (0) | 0/56 (0) | NA |  | | 5.3 ± 2.1 | | 33 | 3.5 ± 1.0 | 38 | <0.001 |
| Week 4 | 4.5 ± 1.9 | 25 | 3.7 ± 1.5 | 48 | 0.056 | |  | 6/25 (24) | 14/48 (29.2) | 0.639 |  | | 5.7 ± 2.1 | | 24 | 3.4 ± 1.2 | 5 | 0.018 |
| Week 8 | 3.6 ± 1.5 | 25 | 3.0 ± 1.2 | 20 | 0.269 | |  | 10/25 (40) | 9/20 (45) | 0.736 |  | | 5.8 ± 2.1 | | 22 | 4.2 ± 1.0 | 2 | 0.117 |
| Week 12 | 3.1 ± 1.3 | 26 | 2.7 ± 0.9 | 48 | 0.109 | |  | 12/26 (46.2) | 29/48 (60.4) | 0.239 |  | | 5.1 ± 2.2 | | 22 | 3.4 ± 1.0 | 4 | 0.136 |
| Week 24 | 2.7 ± 0.8 | 28 | 2.3 ± 0.5 | 52 | 0.089 | |  | 15/28 (53.6) | 40/52 (76.9) | 0.032 |  | | 4.8 ± 2.0 | | 27 | 3.4 ± 0.8 | 30 | 0.004 |
| Week 36 | 2.4 ± 0.5 | 27 | 2.2 ± 0.2 | 49 | 0.005 | |  | 15/27 (55.6) | 42/49 (85.7) | 0.012 |  | | NA | | 0 | NA | 0 | NA |
| Week 48 | 2.2 ± 0.3 | 33 | 2.2 ± 0.3 | 56 | 0.169 | |  | 27/33 (81.8) | 51/56 (91.1) | 0.317 |  | | 4.0 ± 1.9 | | 29 | 3.2 ± 0.9 | 29 | 0.070 |
| Week 96 | 2.2 ± 0.1 | 30 | 2.2 ± 0.2 | 52 | 0.351 | |  | 26/30 (86.7) | 50/52 (96.2) | 0.185 |  | | 3.0 ± 1.4 | | 13 | 1.7 ± 1.9 | 19 | 0.095 |
| Week 144 | 2.2 ± 0.2 | 30 | 2.2 ± 0.2 | 37 | 0.346 | |  | 28/30 (93.3) | 36/37 (97.3) | 0.583 |  | | 1.2 ± 1.0 | | 7 | 2.2 ± 1.0 | 9 | 0.081 |
| Week 192 | 2.1 ± 0.2 | 26 | 2.1 ± 0.0 | 19 | 0.710 | |  | 26/26 (100) | 19/19 (100) | >0.99 |  | | 1.2 ± 1.3 | | 6 | 2.2 ± 1.2 | 7 | 0.199 |
| Week 240 | 2.2 ± 0.2 | 21 | 2.1 ± 0.0 | 7 | 0.300 | |  | 21/21 (100) | 7/7 (100) | >0.99 |  | | 1.3 ± 1.1 | | 7 | 2.8 | 1 | 0.127 |

Results are *n* (%), or mean ± standard deviation.

**Abbreviations:** cLAM, lamivudine; LAM-R, LAM-resistant; LAM-S, LAM-susceptible; HBV, hepatitis B virus; HBsAg, HBV surface antigen; NA, not applicable
