# Supplementary material for: Virological Response to Tenofovir Disoproxil Fumarate in HIV-Positive Patients with Lamivudine-Resistant Hepatitis B Virus Coinfection in an Area Hyperendemic for Hepatitis B Virus Infection
Source: PLoS One. 2016 Dec 29;11(12):e0169228. doi: 10.1371/journal.pone.0169228 (PMC5199102; doi:10.1371/journal.pone.0169228)
Supplement: S3 Table — (DOC) [file pone.0169228.s006.doc]

**Supplementary Table 3.** Response of HBV to tenofovir-containing antiretroviral therapy based on presence of baseline HBeAg positivity

|  | Plasma HBV DNA (log_10_ copies/mL) | | | | | |  | Plasma HBV DNA <128 copies/mL, n (%) | | | |  | | HBsAg level (log_10_IU/mL) | | | | |
| --- | --- | --- | --- | --- | --- | --- | --- | --- | --- | --- | --- | --- | --- | --- | --- | --- | --- | --- |
|  | HBeAg + | n | HBeAg - | n | | *P* |  | HBeAg + | HBeAg - | p |  | | HBeAg + | | n | HBeAg - | n | p |
| Baseline | 7.6 ± 1.6 | 34 | 5.0 ± 1.8 | 54 | <0.001 | |  | 0/34 (0) | 0/54 (0) | NA |  | | 5.6 ± 1.6 | | 28 | 3.5 ± 1.4 | 43 | <0.001 |
| Week 4 | 5.2 ± 1.4 | 31 | 3.0 ± 1.3 | 41 | <0.001 | |  | 1/31 (3.2) | 19/41 (46.3) | <0.001 |  | | 6.4 ± 1.8 | | 17 | 3.9 ± 1.7 | 11 | 0.002 |
| Week 8 | 4.4 ± 1.2 | 20 | 2.6 ± 0.9 | 25 | <0.001 | |  | 1/20 (5) | 18/25 (72) | <0.001 |  | | 6.6 ± 1.5 | | 15 | 4.0 ± 1.9 | 9 | 0.007 |
| Week 12 | 3.8 ± 1.2 | 27 | 2.3 ± 0.4 | 47 | <0.001 | |  | 3/27 (11.1) | 38/47 (80.9) | <0.001 |  | | 6.3 ± 1.5 | | 13 | 3.4 ± 1.5 | 13 | 0.001 |
| Week 24 | 2.9 ± 0.8 | 30 | 2.2 ± 0.3 | 49 | <0.001 | |  | 9/30 (30) | 45/49 (91.8) | <0.001 |  | | 5.1 ± 1.6 | | 24 | 3.3 ± 1.1 | 33 | <0.001 |
| Week 36 | 2.4 ± 0.5 | 32 | 2.1 ± 0.2 | 43 | <0.001 | |  | 15/32 (46.9) | 41/43 (95.3) | <0.001 |  | | NA | | 0 | NA | 0 | NA |
| Week 48 | 2.4 ± 0.4 | 34 | 2.1 ± 0.0 | 54 | <0.001 | |  | 23/34 (67.6) | 54/54 (100) | <0.001 |  | | 4.3 ± 1.7 | | 24 | 3.0 ± 1.1 | 34 | 0.001 |
| Week 96 | 2.2 ± 0.3 | 33 | 2.1 ± 0.0 | 48 | 0.007 | |  | 27/33 (81.8) | 48/48 (100) | 0.003 |  | | 2.8 ± 2.1 | | 12 | 1.9 ± 1.5 | 20 | 0.032 |
| Week 144 | 2.2 ± 0.2 | 28 | 2.1 ± 0.0 | 39 | <0.001 | |  | 25/28 (89.3) | 39/39 (100) | 0.068 |  | | 1.7 ± 1.7 | | 3 | 1.8 ± 1.0 | 13 | 0.946 |
| Week 192 | 2.2 ± 0.2 | 20 | 2.1 ± 0.0 | 25 | 0.048 | |  | 20/20 (100) | 25/25 (100) | NA |  | | 2.1 ± 1.6 | | 4 | 1.6 ± 1.3 | 9 | 0.537 |
| Week 240 | 2.2 ± 0.2 | 15 | 2.1 ± 0.0 | 13 | 0.095 | |  | 15/15 (100) | 13/13 (100) | NA |  | | 1.3 ± 2.0 | | 3 | 1.7 ± 0.6 | 5 | 0.881 |

Results are *n* (%), or mean ± standard deviation.

**Abbreviations:** HBV, hepatitis B virus; HBeAg, HBV envelope antigen; HBsAg, HBV surface antigen; NA, not applicable
