# Supplementary material for: Virological Response to Tenofovir Disoproxil Fumarate in HIV-Positive Patients with Lamivudine-Resistant Hepatitis B Virus Coinfection in an Area Hyperendemic for Hepatitis B Virus Infection
Source: PLoS One. 2016 Dec 29;11(12):e0169228. doi: 10.1371/journal.pone.0169228 (PMC5199102; doi:10.1371/journal.pone.0169228)
Supplement: S4 Table — (DOC) [file pone.0169228.s007.doc]

**Supplementary Table 4.** Change of estimated glomerular filtration rate (eGFR) under tenofovir-containing combination antiretroviral therapy based on presence of lamivudine-resistance to HBV

|  | eGFR (ml/min/1.73m^2^) | | | | |
| --- | --- | --- | --- | --- | --- |
|  | LAM-R | n | LAM-S | n | p |
| Baseline | 97 ± 14 | 33 | 110 ± 25 | 56 | 0.005 |
| Week 48 | 98 ± 16 | 33 | 102 ± 22 | 56 | 0.450 |
| Week 96 | 98 ± 16 | 30 | 104 ± 26 | 52 | 0.260 |
| Week 144 | 99 ± 17 | 30 | 98 ± 26 | 37 | 0.262 |
| Week 192 | 97 ± 16 | 26 | 98 ± 28 | 19 | 0.765 |
| Week 240 | 99 ± 18 | 21 | 93 ± 17 | 7 | 0.474 |

Results are *n* (%), or mean ± standard deviation.

**Abbreviations:** eGFR, estimated glomerular filtration rate; LAM, lamivudine; LAM-R, LAM-resistant; LAM-S, LAM-susceptible
