# Supplementary material for: Virological Response to Tenofovir Disoproxil Fumarate in HIV-Positive Patients with Lamivudine-Resistant Hepatitis B Virus Coinfection in an Area Hyperendemic for Hepatitis B Virus Infection
Source: PLoS One. 2016 Dec 29;11(12):e0169228. doi: 10.1371/journal.pone.0169228 (PMC5199102; doi:10.1371/journal.pone.0169228)
Supplement: S5 Table — (DOC) [file pone.0169228.s008.doc]

**Supplementary Table 5.** Changes of alanine aminotransferase, alpha-fetoprotein, and APRI score in response to tenofovir-containing combination antiretroviral therapy based on presence of lamivudine-resistance to HBV

|  | ALT (IU/L) | | | | | |  | AFP (ng/mL) | | |  | | APRI score | | | | | |
| --- | --- | --- | --- | --- | --- | --- | --- | --- | --- | --- | --- | --- | --- | --- | --- | --- | --- | --- |
|  | LAM-R | n | LAM-S | n | | p |  | LAM-R (n) | LAM-R (n) | p | |  | | LAM-R | n | LAM-S | n | p |
| Baseline | 56 ± 55 | 29 | 52 ± 48 | 52 | 0.383 | |  | 17 ± 39 (26) | 54 ± 257 (28) | 0.910 | |  | | 0.5 ± 0.4 | 33 | 0.9 ± 1.4 | 55 | 0.099 |
| Week 48 | 31 ± 16 | 33 | 30 ± 19 | 41 | 0.535 | |  | 6± 5 (11) | 5 ± 9 (15) | 0.233 | |  | | 0.4 ± 0.2 | 31 | 0.4 ± 0.1 | 43 | 0.869 |
| Week 96 | 27 ± 14 | 25 | 35 ± 25 | 38 | 0.191 | |  | 3 ± 1 (16) | 7 ± 12 (10) | 0.526 | |  | | 0.3 ± 0.1 | 29 | 0.4 ± 0.2 | 39 | 0.399 |
| Week 144 | 27 ± 15 | 21 | 32 ± 15 | 33 | 0.153 | |  | 3 ± 1 (12) | 9 ± 16 (7) | 0.147 | |  | | 0.3 ± 0.1 | 28 | 0.3 ± 0.1 | 26 | 0.822 |
| Week 192 | 28 ± 12 | 25 | 38 ± 23 | 15 | 0.356 | |  | 3 ± 2 (8) | 13 ± 21 (4) | 0.481 | |  | | 0.4 ± 0.2 | 24 | 0.4 ± 0.2 | 13 | 0.504 |
| Week 240 | 28 ± 12 | 20 | 37 ± 12 | 6 | 0.670 | |  | 2.08 (1) | 3 ± 0.6 (4) | 0.157 | |  | | 0.4 ± 0.1 | 20 | 0.3 ± 0.1 | 6 | 0.503 |

Results are *n* (%), or mean ± standard deviation.

**Abbreviations:** AFP, alpha-fetoprotein; ALT, alanine aminotransferase; APRI, AST-to-platelet ratio index; HBV, hepatitis B virus; LAM, lamivudine; LAM-R, LAM-resistant; LAM-S, LAM-susceptible; NA, not applicable

| ALT  Baseline vs. week 48 (n=69), p<0.001  Baseline vs. week 96 (n=60), p=0.001  Baseline vs. week 144 (n=52), p=0.002  Baseline vs. week 192 (n=38), p=0.022  Baseline vs. week 240 (n=25), p=0.049 | AFP  Baseline vs. week 48 (n=20), p=0.261  Baseline vs. week 96 (n=20), p=0.283  Baseline vs. week 144 (n=14), p=0.263  Baseline vs. week 192 (n=9), p=0.168  Baseline vs. week 240 (n=4), p =0.514 | APRI  Baseline vs. week 48 (n=74), p=0.007  Baseline vs. week 96 (n=68), p <0.001  Baseline vs. week 144 (n=54), p<0.001  Baseline vs. week 192 (n=36), p=0.071  Baseline vs. week 240 (n=25), p =0.049 |
| --- | --- | --- |
